# Phenotypic plasticity of pre-adult egg maturation in a parasitoid: Effects of host-starvation and brood size

**DOI:** 10.1371/journal.pone.0195767

**Published:** 2018-04-16

**Authors:** Yael Keinan, Rachel Braun, Tamar Keasar

**Affiliations:** 1 Evolutionary and Environmental Biology, University of Haifa, Haifa, Israel; 2 Human Biology, University of Haifa, Haifa, Israel; 3 Biology and the Environment, University of Haifa–Oranim, Tivon, Israel; Ben-Gurion University of the Negev, ISRAEL

## Abstract

Larvae of parasitoid wasps develop on a single arthropod host, and often face resource limitation that induces a tradeoff between egg maturation and somatic growth. Part of the variation in the growth-reproduction allocation was shown to be heritable, but how the larval developmental environment affects this allocation is not well-known. Detection of life history tradeoffs is often facilitated under stress conditions. We therefore exposed developing female larvae of the polyembryonic parasitoid *Copidosoma koehleri* (Hymenoptera: Encyrtidae) to laboratory manipulations aimed to restrict host resources (either host-starvation or high larval density). We compared the females’ body sizes and egg loads shortly after adult emergence (<24 h) to those of closely related control females, which developed at a lower larval density within non-starved hosts. Host-starvation reduced the females’ body sizes but not their initial egg loads. Females that experienced high larval density produced more eggs but were similar in body size to the low-density controls. Thus, the relative allocation to reproduction increased in response to both manipulations of host condition. Developmental duration and longevity were similar in all treatments. The negative correlation between body size and reproductive allocation, observed in the host-starvation treatment, is compatible with previous evidence from other parasitoids. In the high larval density treatment, however, reproductive allocation increased while body size was maintained, suggesting that the higher density increased rather than limited host resources per developing parasitoid female. The additional host resources that were diverted into egg production possibly resulted from increased feeding and body mass gain by hosts parasitized by large broods of wasps. Our results demonstrate phenotypic plasticity in resource allocation between growth and reproduction in a developing parasitoid. This plasticity may contribute to an adaptive balance between longevity and mobility vs. fecundity during the adult stage.

## Introduction

Animals trade off somatic growth and maintenance with reproductive investment under resource limitations. The tradeoffs are frequently manifested as negative correlations between life-history traits such as fecundity and longevity [1]. Some of the tradeoffs are evolved and heritable, resulting from selection for one life-history trait at the expense of another [2]. In other cases, an individual’s resource allocation to survival vs. reproduction varies with environmental conditions, indicating phenotypic plasticity in balancing the tradeoff [3]. Tradeoffs due to resource constraints often apply to larvae of parasitoid wasps that consume a single host individual during their development. The nutrients obtained from the host are then allocated to the parasitoids’ body tissues or gametes [4]. In most parasitoid species, egg development starts during the larval phase and continues in the adult. The fraction of the egg complement that is mature at adult emergence is termed the Ovigeny Index (OI) and provides a measure of early-life investment in reproduction. The index ranges from 0 to 1. An OI of 1 (strict pro-ovigeny) indicates that the female emerges with all of her oocytes mature and ready for oviposition; whereas an OI of 0 (extreme syn-ovigeny) denotes that the female emerges with no mature oocytes [5].

Past studies have investigated the selective factors that shape resource allocation in developing parasitoids [4, 6]. Life history theory predicts that the optimal timing of reproductive investment by parasitoid females will depend on the local availability of hosts. Host-rich environments are expected to select for early egg production in females, thereby increasing their OI. This would allow the parasitoids to oviposit into hosts as soon as they are encountered, and to avoid fitness loss due to egg limitation. The predicted associated cost is reduced somatic growth, resulting in smaller, less mobile and shorter-lived adults. In host-limited environments, on the other hand, selection is expected to favor larger-sized individuals, with higher longevity and dispersal abilities but with delayed egg production (lower OI) [7].

In agreement with this proposed tradeoff, parasitoids originating from host-rich populations exhibit higher early-life egg production and lower somatic investment than their conspecifics from host-limited habitats. *Asobara tabida* (Hymenoptera: Braconidae) females from southern European populations, where host availability is higher, had larger initial egg loads and lower fat reserves than females from northern populations with fewer hosts [6]. In the pro-ovigenic parasitoid *Anagrus daanei* (Hymenoptera: Mymaridae), egg loads increased with host densities across sampling sites: females from natural habitats with lower host densities produced fewer and larger eggs than females sampled from host-rich agricultural habitats [8]. Small *Aphaereta genevensis* (Hymenoptera: Braconidae) females produced a higher proportion of their eggs as larvae than large females [9], supporting a tradeoff between investment in egg production vs. somatic growth.

For natural selection to affect early-life egg maturation rates, this trait must be associated with heritable variation. This was indeed found in a few studies: Initial egg loads in *Trichogramma brassicae* (Hymenoptera: Trichogrammatidae) showed significant mother-daughter correlations and varied among genetic lines [10]. Total fecundity had significant heritability in *Anagrus delicatus* [11]. As this species is strictly pro-ovigenic, this indicates a genetic component to pre-adult egg maturation. Finally, in the polyembryonic gregarious parasitoid *Copidosoma koehleri*, the focus of the present study, variation in initial egg loads among individuals decreased with genetic relatedness [12].

Nevertheless, most of the variation in the timing of reproductive investment of parasitoids is not explained by genetic background [10, 12]. An additional potential source of variation is pre-maturation phenotypic plasticity, namely adjustment of egg maturation rates to the environmental conditions experienced by developing larvae. Here, we address this possibility by exposing closely related larvae to different developmental environments and recording the egg loads and body sizes of the emerging adults. As life-history tradeoffs are most conspicuous under stress conditions [13], we applied two experimental manipulations intended to limit the resources per developing parasitoid. In one experimental treatment we induced host-starvation, aimed to reduce the total amount of food resources available to the developing parasitoids. In a second treatment we increased brood sizes (the number of wasp larvae developing within the same host) in normally feeding hosts, to increase the number of competitors for the hosts’ resources. We expected the restriction of host resources per developing larva to increase the parasitoids’ need to trade off reproductive with somatic investment, and possibly also to alter the allocation between the two functions.

We expected the manipulations of developmental environment to affect the body size of the emerging females, their initial egg loads, or both parameters. Such effects would confirm that our experimental interventions indeed resulted in resource limitation for the developing parasitoids. If the manipulations of developmental environment affect the parasitoids’ allocation to larval egg maturation vs. somatic growth, this would support phenotypic plasticity in early egg maturation. And if smaller individuals allocate a larger proportion of their resources to egg maturation, this would support the previously demonstrated correlation between body size and ovigeny [4, 9].

## Materials and methods

### The study organism

*Copidosoma koehleri* (Blanchard) (Hymenoptera: Encyrtidae) is a koinobiont, polyembryonic egg-larval endoparasitoid, about 1.5 mm long. It parasitizes the potato tuber moth, *Phthorimaea operculella* (Zeller) (Lepidoptera: Gelechiidae), and is used as its natural enemy for pest management purposes [14–16]. Females produce small eggs that are poor in nutrients. The species has been proposed to be pro-ovigenic [17] (the females’ full egg complement is mature at adult emergence and no additional eggs are produced during the adult stage), but a recent study showed that *C*. *koehleri* Females mature only about 30% of their eggs before adult emergence [12]. The longevity of honey-fed adults is about 30 days under lab conditions [18] and the wasps do not host-feed (personal observation). Superparasitism occurs frequently as one or several females lay more than one egg per host [19]. After emerging from the egg, the host goes through four larval instars while the parasitoid embryos develop inside it. The proliferation of the parasitoid egg is complete within six to ten days after oviposition and results in a clone of approximately 40 genetically identical embryos [19, 20]. After completely consuming the host tissues, leaving only its cuticle, the parasitoid larvae pupate in the host mummy and later emerge as adults. Egg-to-adult development requires ca. 30 days at 27°C. Sex determination is haplo-diploid, i.e., virgin females produce only haploid sons, whereas mated females can produce both haploid sons and diploid daughters [21]. Female clone-members exhibit a larval caste system: a single soldier larva develops precociously and attacks members of competing clones that superparasitize the same host. It dies before reaching maturity [18, 20, 21–22]. The remaining female larvae develop normally and form the reproductive caste. Male clones do not form soldiers.

### Insect rearing

The laboratory stock of *C*. *koehleri* used in the study originated from field-collected individuals from South Africa in 2003. The parasitoids and their hosts were reared using modifications of previous published protocols [16, 23], under controlled conditions of 27°C, 60% relative humidity and a 12:12 h light: dark schedule. Adult parasitoids were fed on honey without restriction. Hosts fed on potato tubers during larval stages, and on honey and water throughout adulthood.

### Obtaining single-sex genetically identical clones

Parasitized mummies were collected from the laboratory stock, placed individually in glass test tubes (13/100 mm) with a drop of honey as food source, and left to emerge. On the day of emergence virgin females (isolated from all-female broods) were mated with a single male, and observed under a stereomicroscope to confirm copulation. The females were then allowed to oviposit individually in ca. 30 fresh (< 24h) *P*. *operculella* eggs for 3–4 hours in a petri dish. No superparasitism is expected under these conditions, as females avoid self superparsitism [24, 25]. Each female's parasitized hosts were placed separately on potato tubers in plastic containers covered with cloth and were reared until pupation. F1 mummies were collected, placed individually in glass test tubes and left to emerge. F1 female clones originating from different mothers and fathers were then used in the experiment. Some of the emerged all-male F1 clones were used to mate non-sister females in the main experiment, as detailed in the “experimental design” section.

### Experimental design

**Fig 1:**
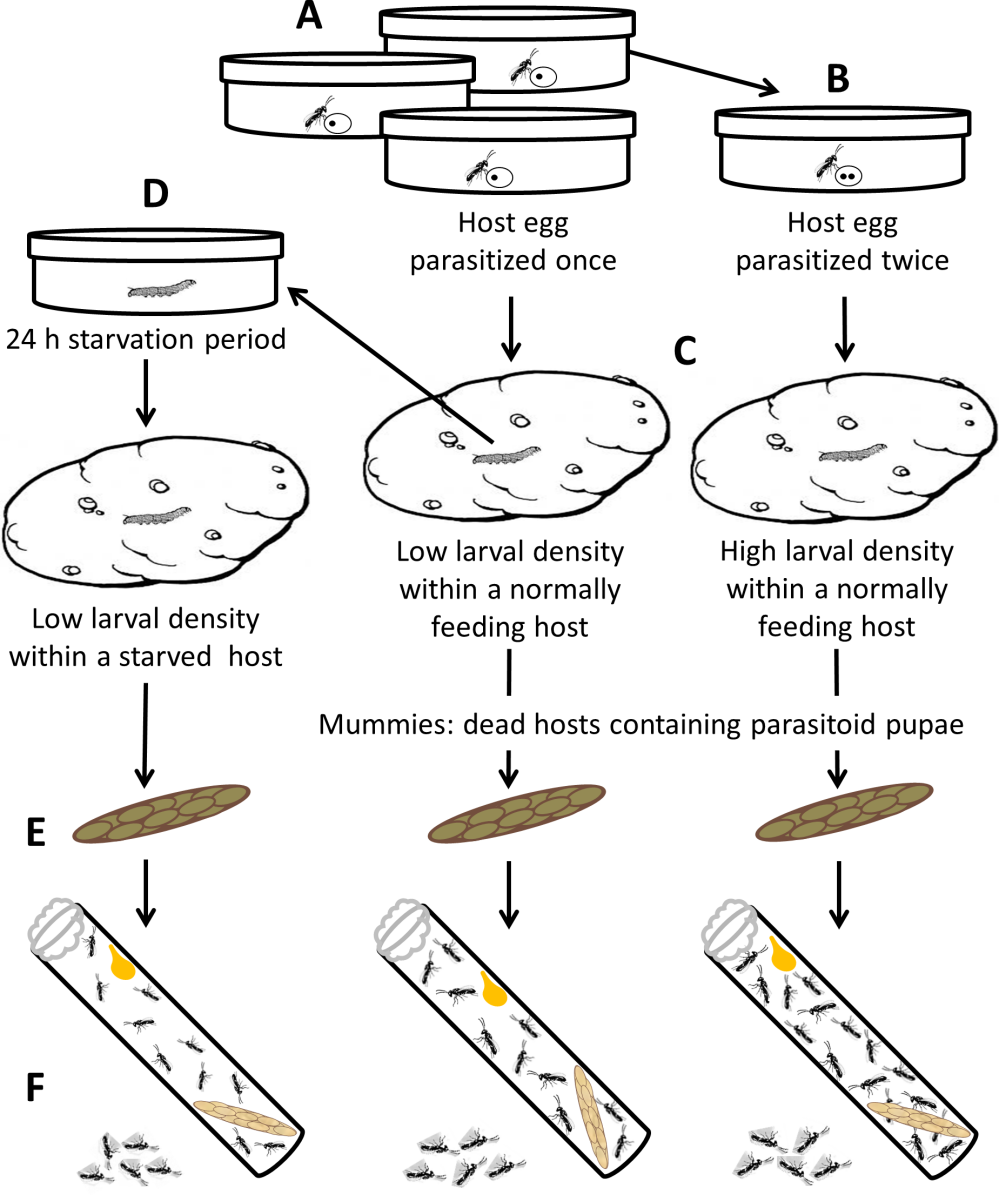
The experimental design. A. Hosts are parasitized once. B. Some of the hosts are parasitized again by a clone-mate female. C. Parasitized hosts are reared on potato tubers. D. 10–12 days later, some of the singly parasitized hosts are excavated out of their tubers and starved for 24 h. E. Mummies (dead hosts containing parasitoid pupae) are collected and reared individually until adult emergence. F. Emergence tubes. On the day of emergence all broods are sexed and counted to ensure their suitability for high or low larval density. 3–5 females from each clone are sacrificed to determine their initial egg loads and hind tibia lengths. The remaining females are kept unmated and supplied with honey *ad lib* to determine per clone average longevity.

Fig 1 provides an overview of the experimental design. In polyembryonic parasitoids, such as our study species, it is straightforward to obtain genetically identical individuals for experiments. This provides the advantage of paired experimental designs when studying environmental effects on phenotypic plasticity: individuals from a single genotype (clone-members) can be exposed to different environments, and the effects on life history traits can be isolated. Fifteen F1 non-sister female clones were obtained as described above. Three mated females from each clone were allowed to oviposit once in five fresh *P*. *operculella* eggs. All ovipositions were observed under a stereomicroscope and the duration of ovipositor insertions were measured to ensure oviposition (ovipositor insertions of >10 seconds reliably indicate oviposition, [24]). Overall fifteen singly parasitized hosts per F1 clone were produced. Ten of the fifteen parasitized hosts per clone were left parasitized once while the remaining five were then introduced to a forth clone-mate female for a second oviposition, yielding hosts parasitized twice. Parasitized hosts were reared until pupation as described above and were separated by parasitizing clone. About 10–12 days later, when they reached the beginning of the fourth larval instar (head width within the range of 877–935 μm [20]), five of the ten singly-parasitized hosts from each clone were excavated out of their tubers and starved for 24 hours in a petri dish with moist cotton wool. This procedure results in host mass loss [25]. The starved parasitized hosts were later placed on new tubers and left to develop until mummified.

Mummies from all three groups were collected, placed individually in glass test tubes (13/100 mm) with a drop of honey as food source, and left to emerge. We aimed for two larval densities within the host: low density of ~45 developing females (consistent with a singly parasitized host) vs. high density of ~65 developing females (consistent with a double-parasitized host [20]). Therefore, on the day of emergence of the adult parasitoid broods, the wasps were sexed and counted to ensure their suitability for one of the three treatments: low larval density in a normally feeding host (LLD, ~45 developing females), low larval density in a starved host (~45 developing females) or high larval density in a normally feeding host (HLD, ~65 developing females). Parasitized hosts designated for one density treatment that yielded unsuitable brood sizes were used for the other treatment according to the number of adult female parasitoids emerging from them. 3–5 females from each brood were sacrificed for measurement and dissection, as detailed below. The remaining females were kept unmated, host-deprived and supplied with honey *ad lib*. The number of surviving wasps per clone was recorded daily and the per-clone average life span was calculated.

### Dissections, egg counting and body size measurements

On the day of emergence, female parasitoids were deep-frozen (-20c°) and individually dissected in a droplet of insect Ringer's solution [26]. Their ovaries were removed and spread out under a light microscope and photographed using a 'Dino-lite' digital eyepiece camera. Images were analyzed using the 'ImageJ' image processing and analysis software [27] to count the number of eggs in each ovary. Hind tibia length was determined for each female, as a measure of body size, using the same equipment.

### Data analysis

We treated offspring clone as the experimental unit of replication and calculated the per-clone mean development times, tibia length, initial egg loads and longevity (n = 15 for each treatment). All distributions, apart from mean development times, conformed to the assumptions of parametric tests. We used linear mixed-effects models with a repeated measures factor to check for differences in the mean per-clone values of these traits and defined the clones’ mother as the subject variable. This was done since sister clones (mummies originating from the same parents, that had developed in different hosts and are 75% genetically identical) were represented in all three treatment groups. Thus, our design is similar to testing each of our subjects across multiple experimental conditions [28].

The Kaplan-Meier estimates of survival in the three treatments, based on per-clone means, were compared using a log-rank test (Package OISurv in R 3.3.2). Correlations between the average life history parameters per clone were calculated using Pearson's coefficient for normally distributed data (tibia length, longevity, initial egg load) and Spearman's rho for data that did not meet parametric assumptions (development time of the HLD treatment group).

We used linear regressions to test for the effect of tibia length on initial egg load within each treatment. We then used ANCOVA, followed by post-hoc tests, to compare among the three regression slopes and intercepts. Egg load, tibia length and treatment were defined as the dependent variable, continuous independent variable and covariate in the ANCOVA, respectively. After finding that egg loads generally increased with body size (see Results), we calculated a “baseline” egg load vs. body size regression for the LLD treatment. Next, we tested whether females in the two other treatments produced similar egg numbers, relative to their body sizes. This was done by computing the residual egg loads of the starvation and HLD wasps, relative to the egg-load vs. tibia length “baseline” regression. We then regressed the residuals on the wasps’ mean per-clone tibia within each treatment. This allowed us to assess whether resource allocation to egg maturation varied with body size relative to the baseline allocation.

SPSS version 19.0 and R version 3.3.2 [29] were used for statistical analyses. The raw data collected in the experiment are provided in S1 File.

## Results

**Fig 2:**
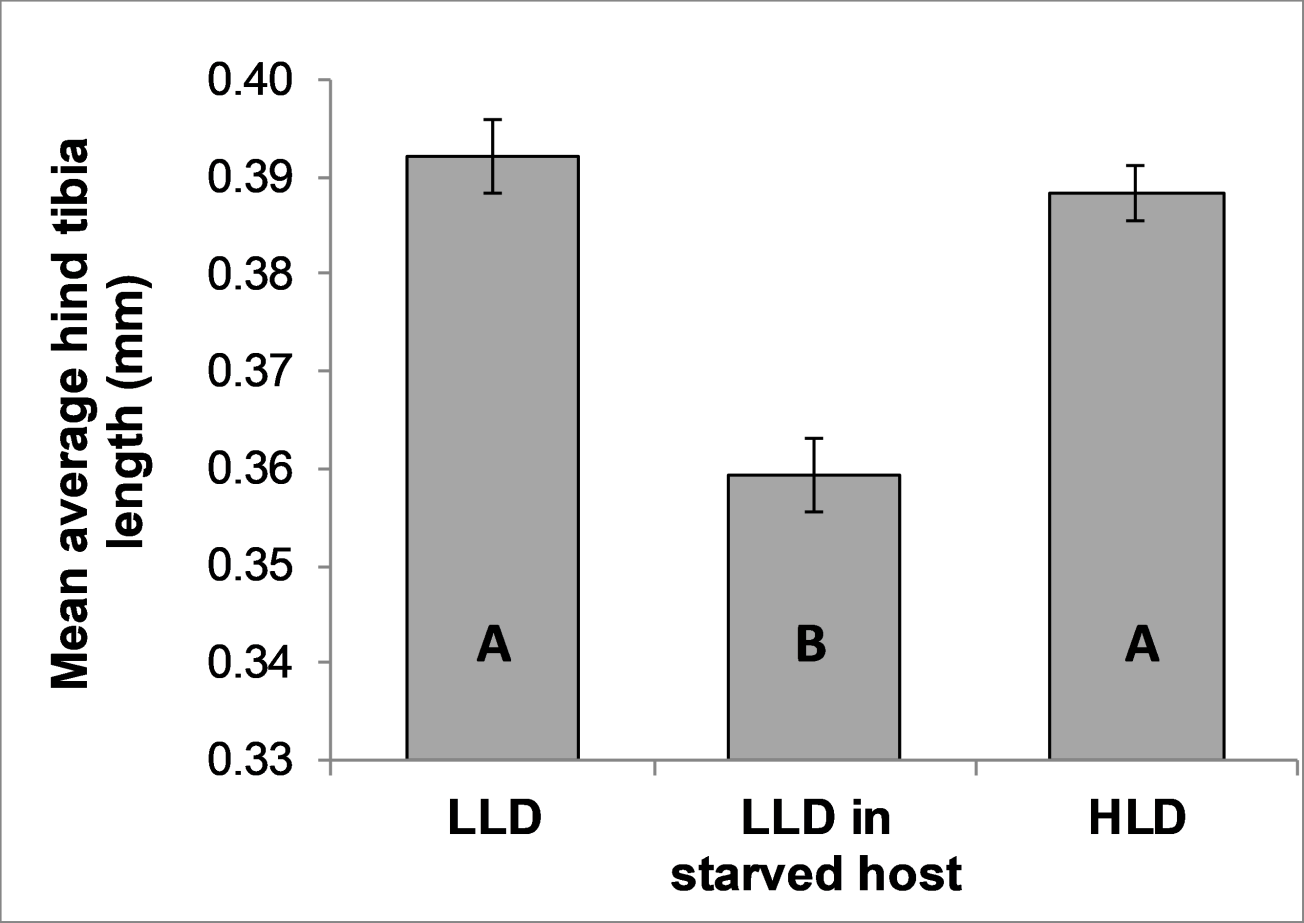
Mean average hind tibia lengths. the average hind tibia length (± SE) of three sister broods developing either at low larval density in a normally feeding host, at low larval density in a starved host or at high larval density in a normally feeding host. Significantly different means in pairwise post-hoc tests are denoted by different letters (P<0.05 for LLD vs. HLD, P<0.001 for HLD vs. starvation).

We aimed for ~45 developing females per host for the LLD and starvation treatments and ~65 developing females per host for the HLD treatment. In good agreement with this plan, the final mean±SD numbers of individuals per brood were 42.29±1.96 in the LLD treatment, 40.93±3.26 in the LLD in starved host treatment and 62.35±3.70 in the HLD treatment. Mean tibia lengths were affected by treatment (repeated-measures ANOVA: F_2,28_ = 26.28, P<0.001). They were lower in the host starvation treatment than in the two non-starvation treatments (Fig 2). The mean duration of egg-to-adult development and mean adult longevity did not differ among treatments (Table 1). The adults’ survival curve was not affected by treatment either (survival analysis: χ_2_ = 0.1, df = 2, P = 0.97, S1 Fig).

**Table 1:** Per-clone development time and longevity.

	LLD	Starvation	HLD	Comparison of means
Development time	32.06±0.34	31.73±0.36	32.37±0.43	F_2, 28_ = 0.65, P = 0.53
Longevity	25.49±2.34	28.52±2.00	26.29±2.49	F_2, 27_ = 1.78, P = 0.19

Mean ±SE per-clone development time and longevity (days). Means were compared using Linear Mixed-Effect models with a repeated-measures factor.

**Fig 3:**
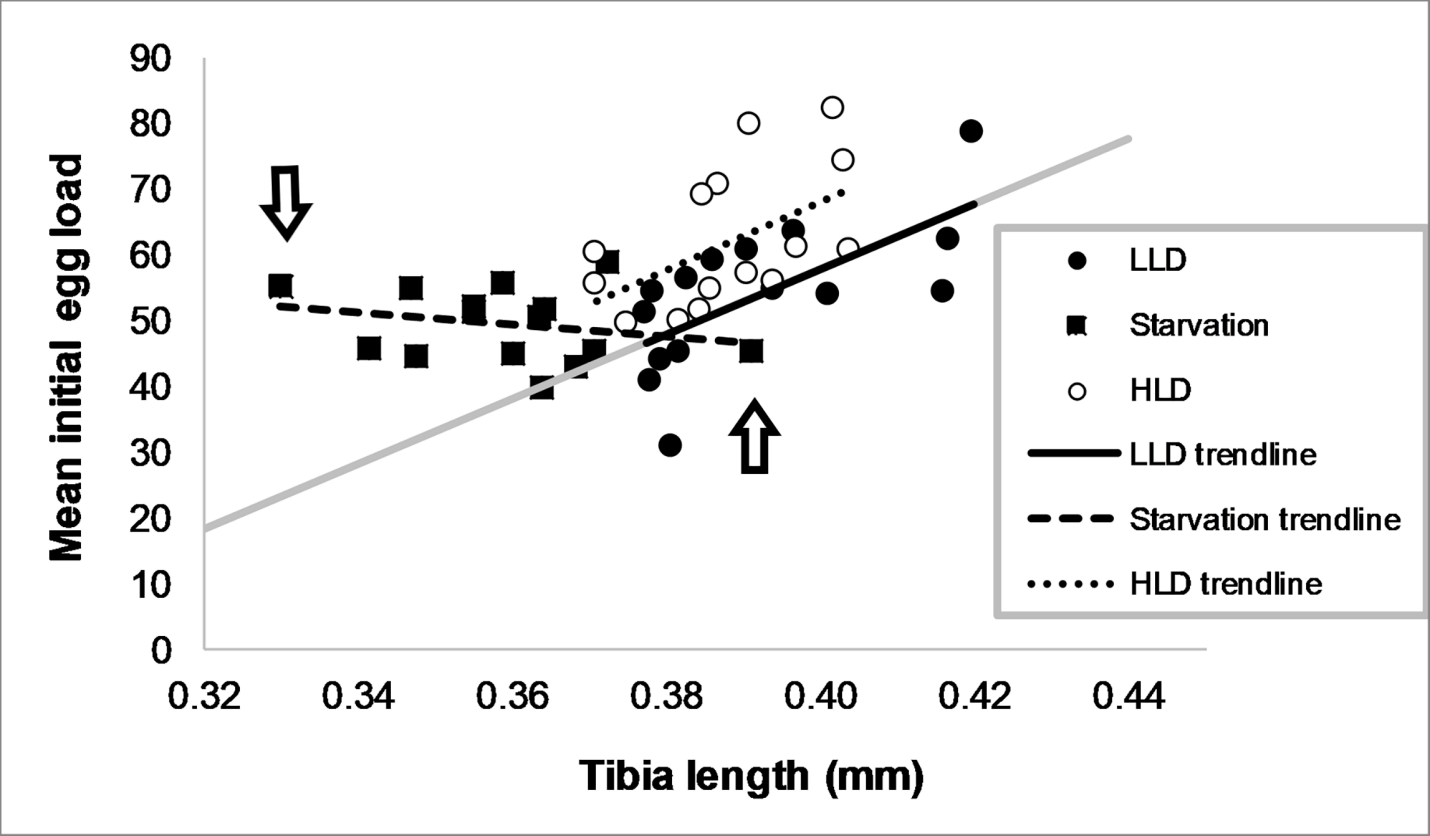
Correlation between body size and initial egg load. Average per-clone hind tibia length vs. average per-clone initial egg load in the three experimental treatments + trend lines. Each data point is an average of three clone-mate females. Each treatment comprised fifteen clones. The “baseline” regression line for the LLD treatment is plotted in gray. Arrows indicate the clones with the smallest and the largest tibia lengths recorded in the starvation treatment.

Per-clone mean initial egg loads were significantly higher in the HLD treatment (mean±SE: 62.12±2.74 eggs) than in the LLD (53.96±2.87 eggs) and starvation (49.49±1.43) treatments (ANOVA followed by Bonferroni post-hoc test, *P*<0.001). They were significantly affected by treatment, by tibia length and by the interaction between these variables (ANCOVA: F_2, 39_ = 5.17, P = 0.01 for treatment, F_1, 39_ = 12.41, P = 0.001 for tibia length, F_2, 39_ = 5.12, P = 0.01 for the interaction). The initial egg loads increased with tibia length within the LLD (Fig 3, Linear regression: Egg load = 500.24×tibia-142.2, *P* = 0.006) and the HLD treatments (Linear regression: Egg load = 515.7×tibia-138.1, *P* = 0.045). In the starvation treatment, on the other hand, initial egg loads showed little variation among clones and were not correlated with tibia length (Linear regression: Egg load = -92.42×tibia+82.69, *P* = 0.385). There were no significant differences in the regression slopes and intercepts between the LLD and the HLD treatments (post-hoc tests: *t* = 0.063, *P* = 1 for slope, *t* = 0.043, *P* = 1 for intercept). The starvation treatment, however, differed from the LLD treatment in both regression parameters (Bonferroni post-hoc tests: *t* = 3.14, *P* = 0.01 for slope, *t* = 3.13, *P* = 0.01 for intercept)). This indicates that more eggs, relative to body size, developed on average in the starvation treatment than in the LLD treatment. In addition, the deviations (residuals) of egg loads from the baseline decreased with tibia length within the starvation treatment (residual = -592.661 ×tibia+224.892, F_1,13_ = 33.289, P<0.001). This is illustrated in Fig 3, where the clones with the smallest and largest tibia lengths of the starvation treatment are marked by arrows. The egg load of the smallest wasps is much higher than the “baseline” trend line of the LLD treatment, while the egg load of the largest wasps falls below the baseline. In other words, smaller wasps within the starvation treatment matured more eggs, relative to their size, than the larger individuals. The residuals of the HLD treatment were not affected by tibia length (residual = 15.458×tibia+4.093, F_1,13_ = 0.004, P = 0.948).

## Discussion

The rate at which eggs mature in the ovaries is a potential limiting factor to insect reproduction, and thus a key process in their life history. Holometabolous insects that are partially pro-ovigenic start producing ovarioles and maturing eggs as pupae [30] and continue oogenesis as adults. Egg maturation requires metabolic resources that are carried over from the larval stage. Energy budget calculations, which use the parasitoid *Venturia canscens* as a model, estimate that only ~10% of the energy accumulated by the larvae is carried over to the adult stage [31]. These low levels of resources underlie the tradeoff between somatic growth and reproduction. They may also constrain egg production, even when the eggs are nutrient-poor and presumably metabolically low-cost, as in our study species.

Evidence for phenotypic plasticity in egg maturation rates in adult parasitoids is abundant. It most commonly involves positive effects of adult nutrition, host availability, and optimal temperature on egg production ([4], but see [32] for a counter-example). Phenotypic plasticity in pre-adult egg production, on the other hand, is much less documented. In one case study, low-temperature stress at the pupal stage enhanced pre-adult egg maturation in *Aphidius ervi* (Braconidae), an aphid parasitoid [33]. A second research study, which focused on fruit-fly parasitoids, found that reproductive allocation in *Diachasmimorpha longicaudata* (Braconidae) varied with the food supplied to its host and hence with host resources. In three other braconid parasitoids of the same host, however, reproductive investment was not affected by the host’s diet [34]. Our experiment provides further evidence for a flexible allocation to egg production vs. growth during pre-adult development.

Females that developed in starved hosts were smaller in size than LLD females from non-starved hosts, in line with earlier findings from *C*. *bakeri* [35]. Initial egg loads, on the other hand, did not differ between the two treatment groups. As brood sizes and egg to adult development times were similar, this suggests that host-starvation indeed reduced the resources available per developing parasitoid larva (and possibly also reduced nutrient quality), and that they allocated a larger proportion of the nutrients to egg maturation. This trend also occurred within the starvation treatment, as small individuals invested more in egg production, relative to their body size, than large individuals. Similarly, pre-adult egg maturation in *A*. *ervi* parasitoids that developed under cold-storage stress was more severely disrupted in large females than in small ones. This was attributed to the lower metabolic requirements of the small individuals [33] and may also explain their relatively higher reproductive allocation in our experiment. Another possible explanation is that *C*. *kpehleri* females always develop a minimum number of eggs regardless of their body size, reflected in the starvation treatment group, with the capacity to develop more if conditions permit. Negative correlations between body size and early egg maturation were also reported in non-manipulative studies, both in inter-specific comparisons [36] and at the intra-specific level [9]. The developmental mechanisms underlying these correlations are still unknown. On the other hand, the undisrupted initial egg load of the starvation treatment females might have been traded off against the quality of the eggs themselves or against the females' ability to produce additional eggs during their adulthood. Further investigation is required to test the viability of these eggs and their capacity to produce offspring clones.

Parasitoids that experienced HLD conditions shared food resources with more individuals than the LLD individuals. In spite of this, HLD wasps had similar body sizes as females from the LLD treatment, but their initial egg loads were higher. Thus, it seems that the total amount of resources per individual was not lower in the HLD treatment than in the LLD control. This is contrary to our initial expectation: we assumed that high larval density would increase the number of competitors for a fixed host resource, and thereby decrease nutrient availability for each competitor. Our experimental design aimed to maximize this resource competition in two ways. First, we induced double-parasitism by genetically identical individuals, whose offspring show low aggression to each other, so as to minimize larval mortality [19]. Second, we dissected and measured females from large broods only (>60 emerged adults) in the HLD treatment. Nevertheless, our aim of creating resource restriction for the developing larvae was not achieved. A possible explanation is that HLD hosts grew larger than LLD ones, providing more nutrients per parasitoid in the HLD treatment. An increase in the mass of HLD mummies, compared to LLD ones, was indeed documented in *C*. *koehleri* [19]. The higher number of eggs matured in the HLD treatment thus reflects increased allocation towards reproduction, although the limitation on host resources is not increased. This begs the question why body size did not increase as well, since large size often confers fitness benefits [35, 37]. Possibly, further somatic growth of the wasps is prevented by physiological constraints, by the associated cost of longer development time, or by a tradeoff with adult survival [13]. Another possibility is that signals indicating a large brood of wasps (whether produced by the host or by the parasitoids) have stronger effects on egg maturation than on somatic growth. Early egg production by parasitoids is often correlated with reduced adult longevity, presumably an additional aspect of the reproduction-survival tradeoff [5, 9]. The use of lipids for egg formation was proposed to limit adult survival, as most adult parasitoids do not synthesize lipids [38]. In the present study, however, there were no differences in adult longevity among the three treatment groups. Energy constraints for females in our experiment may have been alleviated because we allowed them unlimited access to honey. This assumption is supported by the lack of correlation between initial egg loads and adult survival in other experiments that provided adult parasitoids with food [12, 39]. Moreover, all females in our study were host-deprived and thus none of them became egg-limited. However, we speculate that under conditions of unlimited access to hosts (leading to egg limitation), a reproduction-survival tradeoff may be revealed. Having a smaller body size, females from the starvation treatment that would reach egg-limitation might encounter greater difficulty producing and laying more eggs, and possibly pay a price through reduced life span. This possibility is supported by the finding that host-deprived females have a significantly longer life span than their host-exposed twin sisters [12]. Thus, the energetic costs of maturing and laying numerous eggs seem to reduce survival. In addition, the undisrupted longevity of the starvation treatment females may result from egg resorption under conditions of time (host) limitation. To our knowledge there is no direct demonstration of egg resorption in *C*. *koehleri*. However, previous findings support this possibility, as egg loads of host-deprived females initially increased but later decreased by the time of natural death [12].

In conclusion, our results demonstrate that environmental conditions influence the allocation of resources between growth and reproduction in a developing parasitoid. This phenotypic plasticity is probably adaptive in optimizing the balance between longevity and mobility vs. fecundity during the adult stage.

## Supporting information

S1 Fig Kaplan-Meier estimates of survival. The daily percentage of surviving broods in the three experimental treatments. Each data point represents the percentage of broods that are still alive, based on the average per clone life span values.
(TIF)

S1 File "S1 File.xls", experimental data. (XLS)
