# Supplementary figures and images for: Phenotypic plasticity of pre-adult egg maturation in a parasitoid: Effects of host-starvation and brood size
Source: PLoS One. 2018 Apr 16;13(4):e0195767. doi: 10.1371/journal.pone.0195767 (PMC5901773; doi:10.1371/journal.pone.0195767)

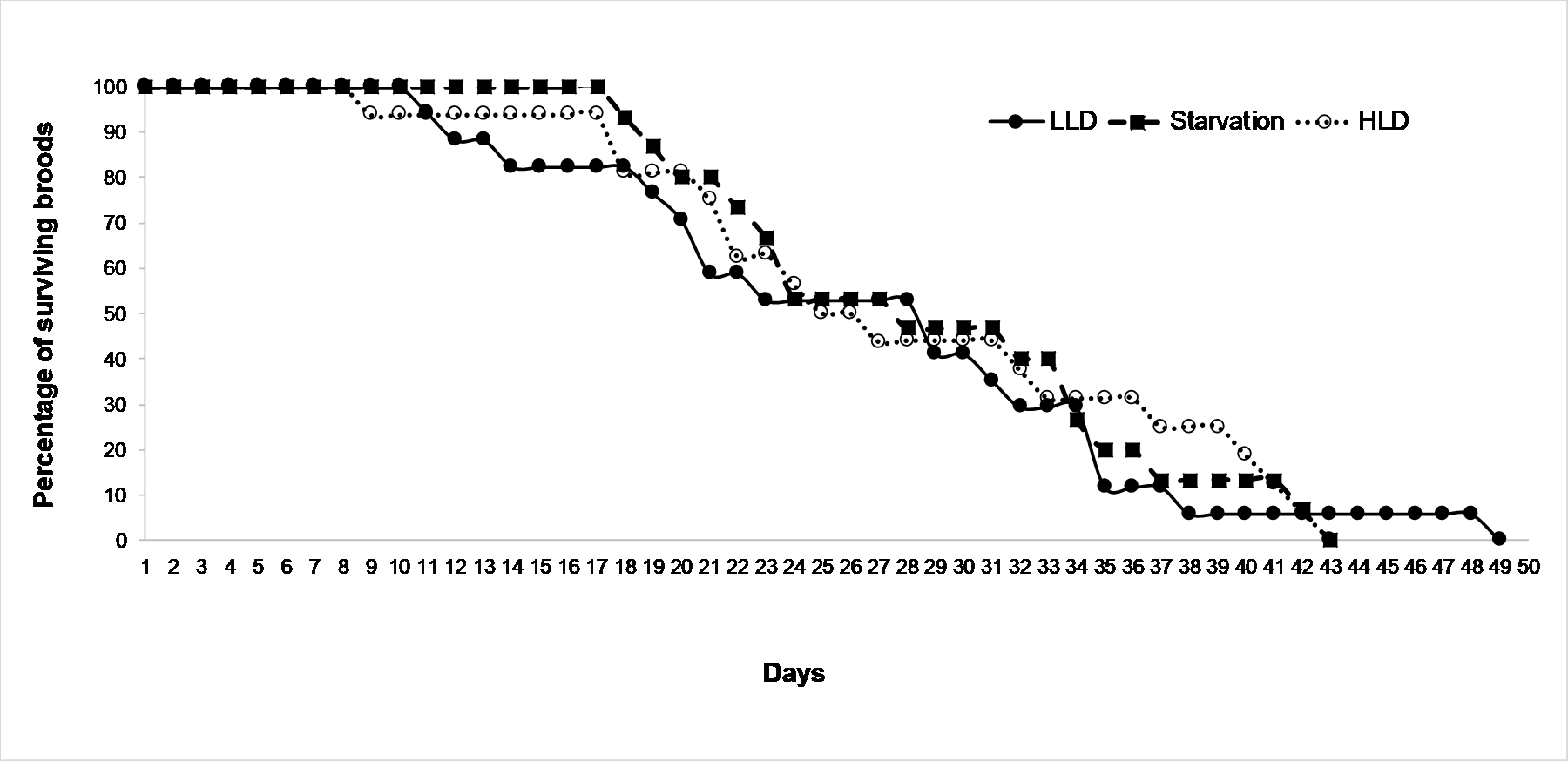

Supplement: S1 Fig — The daily percentage of surviving broods in the three experimental treatments. Each data point represents the percentage of broods that are still alive, based on the average per clone life span values. (TIF) [file pone.0195767.s001.tif]
